# Growth Performance, Health Parameters, and Blood Metabolites of Dairy Calves Supplemented with a Polyherbal Phytogenic Additive

**DOI:** 10.3390/ani15040576

**Published:** 2025-02-17

**Authors:** Lucero Abigail Velázquez-Cruz, German David Mendoza-Martínez, Pedro Abel Hernández-García, Enrique Espinosa-Ayala, Cesar Díaz-Galván, Gabriela Vázquez-Silva, José Felipe Orzuna-Orzuna, Pablo Benjamín Razo-Ortíz, María Eugenia de la Torre-Hernández

**Affiliations:** 1 Centro Universitario Amecameca, Universidad Autónoma del Estado de México, Amecameca CP 56900, Mexico; lucemvz14@gmail.com (L.A.V.-C.); enresayaa1@hotmail.com (E.E.-A.); mvzrazo@gmail.com (P.B.R.-O.); 2 Departamento de Producción Agrícola y Animal, Universidad Autónoma Metropolitana—Xochimilco, Mexico City CP 04960, Mexico; gmendoza@correo.xoc.uam.mx (G.D.M.-M.); cesarwardi14@gmail.com (C.D.-G.); 3 Departamento del Hombre y su Ambiente, Universidad Autónoma Metropolitana—Xochimilco, Mexico City CP 04960, Mexico; gavaz@correo.xoc.uam.mx; 4 Departamento de Zootecnia, Universidad Autónoma Chapingo, Chapingo CP 56230, Mexico; jforzuna@gmail.com; 5 CONAHCYT-UAM Xochimilco, Universidad Autónoma Metropolitana—Xochimilco, Mexico City CP 04960, Mexico; mdelatorre@correo.xoc.uam.mx

**Keywords:** feed plant additive, polyherbal mixture, blood biochemistry, hematological profile, hydrolyzable tannins, flavonoids

## Abstract

This study aimed to evaluate the effects of dietary supplementation with increasing levels of a polyherbal phytogenic additive (PPA) on growth performance, health parameters, and blood metabolites of dairy calves. Forty female Holstein calves (43.35 ± 2.41 kg body weight and 25.1 ± 2.2 d of age) were housed in individual pens and randomly assigned to four treatments (*n* = 10) with increasing levels of PPA: 0 (CON), 2 (PPA2), 3 (PPA3), and 4 (PPA4) g PPA/d for 67 d. Compared to the CON treatment, supplementation with the PPA2 treatment increased (quadratic effect *p* = 0.02) the average daily gain and final body weight of the dairy calves. Also, a higher starter intake (SI), and intake of milk + milk replacer was observed (linear and quadratic effect *p* = 0.0001) in response to supplementation with the PPA2, PPA3, and PPA4 treatments. The evaluated treatments did not affect (*p* > 0.05) the feed conversion ratio, or the number of cases of diarrhea, pneumonia, and otitis. The evaluated treatments did not affect (*p* > 0.05) most of the hematological and biochemical blood parameters. However, compared to the CON treatment, supplementation with the PPA2 treatment increased (quadratic effect *p* < 0.05) the blood concentration of the segmented neutrophils and plasma protein. Likewise, the serum glucose concentration decreased (linear effect *p* < 0.05) with supplementation of the PPA2, PPA3, and PPA4 treatments. Compared with the CON treatment, the PPA4 supplementation decreased the serum urea concentration (linear effect *p* = 0.04). In conclusion, supplementation with 2 g/d of PPA can be used as a natural alternative to improve the growth rate and stimulate starter and milk + milk replacer intake in dairy calves without adverse effects on health parameters or blood metabolites.

## Simple Summary

Dietary supplementation with polyherbal phytogenic additives has shown positive effects on growth performance and health status in dairy and finishing ruminants. However, there is little information on the effects of polyherbal phytogenic additive supplementation in dairy calves. This study aimed to evaluate the effects of a polyherbal phytogenic additive on the growth performance, health parameters, and blood metabolites of dairy calves. Dietary supplementation with low doses (2 g/d) of the evaluated polyherbal phytogenic additive improved the daily weight gain, final body weight, starter intake, and milk + milk replacer intake without affecting the disease incidence.

## 1. Introduction

In dairy calves, one of the main causes of mortality and economic losses is the incidence of diseases, among which respiratory (mainly pneumonia) and enteric diseases are the most common [1]. In addition, these diseases negatively affect the feed intake and weight gain and increase the amount of antibiotics applied to the animals to cure them [2]. In dairy calves, a low feed intake (milk and milk replacer) and low weight gain in the pre-weaning stage result in lower milk production during the first lactation period [3]. On the other hand, the use of high amounts of antibiotics to cure diseases in dairy calves increases the risks of bacteria resistant to their effects [4]. Therefore, in recent years, there has been increasing interest in the validation of new products of natural origin that improve the milk intake, milk replacer intake, and weight gain in dairy calves and, at the same time, reduce the incidence of diseases and the use of antibiotics [5]. According to Wickramasinghe et al. [6], phytogenic additives are one of the natural products with the potential to reduce the incidence of diseases and improve growth performance in dairy calves.

Phytogenics (also called phytochemicals or phytobiotics) are plant-derived products containing one or more bioactive compounds with therapeutic properties, such as antimicrobial, antioxidant, or immunostimulant effects [7]. Polyherbal phytogenic additives (PPAs) are a particular type of phytogenic, which are composed of mixtures of plant parts and contain several bioactive compounds [8,9]. Several recent studies [9,10,11] show that supplementation with increasing doses (1 to 5 g/d) of some PPAs has beneficial effects on the milk + milk replacer intake and weight gain in dairy calves and, at the same time, decreases the incidence of diarrhea, pneumonia, otitis, and the doses of antibiotics applied to the animals. Among the PPAs currently available is ImmuPlus^®^, which is a commercial product made from plant parts of *Azadirachta indica*, *Tinospora cordifolia*, *Whitania somnífera*, *Andrographis paniculata,* and *Ocimum sanctum*, which contains 12% hydrolyzable tannins [12] and 8.5% flavonoids [13].

The effects of dietary supplementation with hydrolyzable tannins and flavonoids have been studied mainly in ruminants. For example, dietary supplementation with hydrolyzable tannins improves the energy utilization efficiency and growth rate in growing ruminants [12,14]. Likewise, the intake of hydrolyzable tannins improves the nutrient digestibility, immune response, and renal function in adult ruminants [15,16]. On the other hand, two recent meta-analyses [17,18] showed that dietary supplementation with flavonoids improves the feed intake, weight gain, feed efficiency, antioxidant status of blood serum, and serum levels of immunoglobulins A, G, and M in sheep, goats, beef cattle, and dairy cows.

Particularly in dairy calves, dietary supplementation with hydrolyzable tannin extracts improves weight gain and feed efficiency [19,20]. Likewise, supplementation with hydrolyzable tannins decreases the incidence and duration of enteric and respiratory diseases in dairy calves and, at the same time, improves hematological parameters [21]. Other studies [22,23] show that the oral administration of hydrolyzable tannins prevents diarrhea in dairy calves and decreases the recovery time, as well as antibiotics applied to sick animals. On the other hand, Yaghoubi et al. [24] reported that dietary supplementation with flavonoids has positive effects on the feed intake, weight gain, and serum concentration of immunoglobulins G and M in dairy calves. Other authors [25,26] have observed a greater weight gain, feed efficiency, and ruminal concentration of total volatile fatty acids and propionate in pre-weaning dairy calves supplemented with flavonoids. In addition, dietary supplementation with flavonoids improves the activity of digestive enzymes in the small intestine and decreases oxidative stress in the blood of dairy calves [26]. Likewise, the incidence of diarrhea in dairy calves decreases with dietary supplementation of flavonoids [25,27].

Based on the above background, we hypothesize that supplementation with a polyherbal phytogenic additive containing hydrolyzable tannins and flavonoids will have a positive synergistic impact on dairy calves’ growth performance, health parameters, and blood metabolites. Therefore, this study aimed to evaluate the effects of supplementation with a polyherbal phytogenic additive containing hydrolyzable tannins and flavonoids on the growth performance, health parameters, and blood metabolites of dairy calves.

## 2. Materials and Methods

### 2.1. Experimental Location

The experiment was conducted on a commercial dairy farm in Torreon, Coahuila, Mexico (25°39′14.4″ N and 103°27′27.8″ W; altitude: 1139 masl). All calf care procedures were approved by the Research Ethics and Bioethics Committee of the Universidad Autónoma del Estado de México (Protocol #08-10 2019).

### 2.2. Growth Performance and Health Parameters

Forty female Holstein calves (43.35 ± 2.41 kg body weight (BW) and 25.1 ± 2.2 d of age) were housed in individual pens and randomly assigned to four treatments (*n* = 10) with increasing levels of polyherbal phytogenic additive (PPA): 0 (CON), 2 (PPA2), 3 (PPA), and 4 (PPA) g PPA/d for 67 d. The PPA used in the current study was ImmuPlus^®^ (Nuproxa S. de RL. de CV. Querétaro, Mexico), which is a commercial PPA composed of parts of *Whitania somnifera*, *Andrographis paniculata*, *Tinospora cordifolia*, *Ocimum sanctum*, and *Azadirachta indica*. According to the manufacturer’s label, ImmuPlus^®^ is standardized to contain mainly hydrolyzable tannins (120 g/kg of product) [12]. However, Cecchini et al. [13] reported that ImmuPlus^®^ also contains 8.5% of total flavonoids. The intermediate dose (3 g/d) of PPA was chosen based on the results of a previous experiment, in which this dose of the same PPA (ImmuPlus^®^) improved feed and energy efficiency in young lambs [12]. However, other PPAs with a similar composition to the one evaluated in the current study have dose-dependent effects [9,10]. Therefore, the present study included a lower (2 g/d) and a higher (4 g/d) dose of PPA. The upper dose of 4 g/d was chosen based on the results reported by Dorantes-Iturbide et al. [12], who found that, at this dose (4 g/d) of the same PPA evaluated in the current study, growth performance begins to decline. The PPA doses were administered mixed with gelatins orally, following the procedures described by other authors [9,10,11]. For this, gelatin solutions were prepared 24 h prior to use. These gelatins were prepared by dissolving 50 g of gelatin (Coloidales Duché, S.A. de C.V. Mexico City, Mexico) per liter of water. The liquid gelatin was placed in 100 mL glasses, and the corresponding dose of PPA was added to each glass. Finally, the gelatins were refrigerated for 24 h until use. The gelatins were administered daily at 6:00 h orally to each dairy calf.

Dairy calves were housed in individual pens with a surface area of 2.5 m^2^, which had a waterer and steel feeders for milk and concentrate. Before the start of the experimental phase (from day 1 of age), dairy calves were fed at 7:00 am and 5:00 pm every day with a mixture (4 L/feed) of 56% milk and 44% of a non-medicated commercial milk replacer (Premium 22-15, Grupo Nu-3^®^, Guanajuato, Mexico). In addition, from the third day of age of the dairy calves, a commercial starter concentrate was offered (Premium Early Weaning Starter, Nuplen^®^, Durango, Mexico), which was gradually increased and offered ad libitum. Feeding of milk and milk replacer was reduced to once daily from the beginning of the experimental phase (day 25 of age) because the intake of starter concentrate had increased. The milk replacer used came from the same batch and, according to the manufacturer’s label, contained crude protein (22.0%), fat (15.0%), nitrogen-free extract (52.9%), ash (6.0%), moisture (4.0%), and crude fiber (0.1%). The milk replacer was prepared using 130 g/L of water at 65 °C and offered to dairy calves at 39 °C using steel buckets. On the other hand, all the starter concentrate used also came from the same batch and, according to the manufacturer’s label, contained crude protein (21.5%), nitrogen-free extract (47.0%), moisture (13.0%), crude fiber (8.0%), ash (7.0%), and fat (3.0%).

On day 25 of the experiment, all calves were vaccinated against *Clostridium* sp. (2.0 mL/animal, Covexin^®^ 10, MSD Labs, Mexico City, Mexico). Each dairy calf was weighed individually at the beginning (day 25 of age) and at the end (day 92 of age) of the experimental phase to estimate the average daily gain (kg/d) and final body weight (kg). To evaluate the growth and development of the calves, at the end of the experimental phase, height at the withers, height at the hip, and thoracic girth were measured, as described in recent studies [10,11]. On the other hand, daily milk + milk replacer intake (L/d) and starter concentrate intake (kg/d) was measured and recorded daily. The feed conversion ratio was estimated considering the total kg of dry matter consumed (including milk and starter concentrate) divided by the kg of body weight gain of each of the dairy calves, as reported by Díaz et al. [10] and Sánchez et al. [9]. The feed intake variation between individuals between days was evaluated as an indicator of stability [9,12]. Likewise, dairy calves were evaluated daily after morning feeding (between 06:00 and 10:00 h) for pneumonia, otitis, diarrhea, and other diseases, as reported by Lee-Rangel et al. [11]. In addition, the doses of antibiotics applied to sick calves were recorded daily. Antibiotics used to treat diarrhea were gentamicin sulfate (1 mL/kg BW; Dyscural Rumiante^®^, Chinoin Labs, Mexico City, Mexico), while pneumonia and otitis were treated with enrofloxacin (1 mL/kg BW; Fortius LA^®^, Virbac Labs, Guadalajara, Mexico). Dairy calves were weaned after obtaining blood samples and the final BW.

### 2.3. Blood Metabolites

Before the morning feeding (6:00 h) on day 50 of the experimental phase, two blood samples were taken from the jugular vein of each dairy calf. One of the two blood samples (5 mL/dairy calf) was obtained with tubes containing EDTA anticoagulant (BD Vacutainer^®^ K2, Dickinson and Company, Franklin Lakes, NJ, USA). This blood sample was evaluated with an EasyVet^®^ hematology analyzer (QS Kontrolab, Hamburg, Germany) to determine differential leukocyte count, hematocrit, and complete blood count, as recently reported by Sánchez et al. [9]. On the other hand, the other blood sample (5 mL/dairy calf) was obtained with anticoagulant-free tubes (BD Vacutainer^®^, Dickinson and Company, Franklin Lakes, NJ, USA). These blood samples were centrifuged at 3500 rpm for 20 min to obtain blood serum, which was evaluated with an EasyVet^®^ autoanalyzer (QS Kontrolab, Hamburg, Germany) to determine the serum concentration of total protein, albumin, globulin, glucose, β-hydroxybutyrate, cholesterol, urea, creatinine, uric acid, bilirubin, aspartate aminotransferase, alkaline phosphatase, lactate dehydrogenase, calcium, and phosphorus, as recently reported by other authors [9,10,12].

### 2.4. Statistical Analysis

Before performing the statistical analyses, the normality of the data was verified with the Shapiro–Wilk test through the PROC UNIVARIATE procedure of the Statistical Analysis System (SAS) statistical software (v6.4) [28]. Subsequently, the data were analyzed using a completely randomized experimental design through the PROC GLM procedure of SAS, considering each dairy calf as an experimental unit. The initial body weight was initially tested as a covariate for the productive performance data. However, this covariate was not considered in the final statistical model because it was not significant (*p* > 0.05). The final statistical model used was the following:

Y_ij_ = µ + T_i_ + e_ij_

where Y_ij_ represents the observations, µ = overall mean, T_i_ = fixed effect of the i-th treatment, and e_ij_ = random error.

Polynomial orthogonal contrasts were applied to all response variables to assess the linear and quadratic effects of PPA inclusion levels (0, 2, 3, and 4 g/d), as previously reported by other authors [9,10,11]. Significant differences were declared when *p* ≤ 0.05.

## 3. Results

### 3.1. Growth Performance and Health Parameters

Table 1 shows that, compared to the CON treatment, supplementation with the PPA2 treatment increased (quadratic effect *p* = 0.02) the average daily gain (ADG) and final body weight (FBW). Likewise, a higher starter intake (SI), and intake of milk + milk replacer was observed (linear and quadratic effect *p* = 0.0001) in response to supplementation with the PPA2, PPA3, and PPA4 treatments. On the other hand, none of the evaluated treatments affected (*p* > 0.05) the feed intake variation (FIV) between the calves, feed conversion ratio (FCR), final withers height (FWH), final hip height (FHH), final thoracic girth (FTG), number of diarrhea cases, number of pneumonia cases, number of otitis cases, and antibiotic doses applied in sick calves.

### 3.2. Blood Metabolites

Supplementation with increasing levels of PPA (PPA2, PPA3, and PPA4) had no significant effects (*p* > 0.05) on blood levels of hematocrit, hemoglobin, red blood cells, mean corpuscular volume, mean corpuscular hemoglobin, mean corpuscular hemoglobin concentration, platelets, leukocytes, lymphocytes, monocytes, band neutrophils, and basophils (Table 2). Compared to the CON treatment, supplementation with the PPA2 treatment increased (quadratic effect *p* < 0.05) the blood concentration of segmented neutrophils and plasma protein.

The serum glucose concentration decreased (linear effect *p* < 0.05) with PPA2, PPA3, and PPA4 supplementation (Table 3). Also, compared with the CON treatment, PPA4 supplementation decreased (linear effect *p* = 0.04) the serum urea concentration. However, dietary supplementation with the PPA2, PPA3, and PPA4 treatments did not affect (*p* > 0.05) the serum concentration of β-hydroxybutyrate, uric acid, creatinine, total protein, albumin, globulin, albumin/globulin ratio, cholesterol, bilirubin, aspartate aminotransferase, alkaline phosphatase, lactate dehydrogenase, calcium, and phosphorus.

## 4. Discussion

### 4.1. Growth Performance and Health Parameters

In the current study, supplementation with 2 g/d of PPA increased the ADG and FBW, probably due to the observed increase in the SI and milk + milk replacer intake in the calves supplemented with this dose (2 g/d) of PPA. These results suggest that low doses (2 g/d for 67 days) of the evaluated PPA can shorten the age at weaning in dairy calves. Furthermore, the higher ADG in the pre-weaned dairy calves is beneficial because it positively correlates with milk production in the first lactation of the adult cow [3]. In a similar dose–response study, Serri et al. [21] also observed an increased ADG and FBW in dairy calves supplemented with low doses (2 g/d for 70 days) of hydrolyzable tannins. Similarly, in another dose–response study, Yaghoubi et al. [24] reported an increased ADG and FBW in dairy calves supplemented with low doses (0.3 g/d for 60 days) of flavonoids. However, in the studies by Serri et al. [21] and Yaghoubi et al. [24], the mean values of the ADG and FBW decreased as the dose of hydrolyzable tannins or flavonoids increased, which is consistent with the quadratic effects observed in the ADG and FBW values in the current study.

According to Dorantes-Iturbide et al. [12], low doses (1 g/kg DM for 56 days) of the same PPA (ImmuPlus^®^) used in the current study increase (+23.2%) the efficiency of the net energy utilization for weight gain in lambs. In dairy calves, dietary supplementation with hydrolyzable tannins (4 to 6 g/kg DM) increases (5.1 to 14.0%) the digestibility of crude protein, neutral detergent fiber, and organic matter [20]. Likewise, dietary supplementation with flavonoids (3 g/d) increases the digestive enzyme activity in the abomasum (+39.8% protease) and small intestine (+50.6% α-amylase, +75.2% lactase, and +14.2% lipase) of dairy calves [26]. These effects of hydrolyzable tannins and flavonoids contained in the PPA evaluated in the current study could lead to greater absorption and metabolic availability of energy, amino acids, and other nutrients required to form body tissues, which would explain the higher ADG and FBW observed in the dairy calves supplemented with low doses (2 g/d) of PPA. Furthermore, hydrolyzable tannins and flavonoids improve the ADG and FBW in dairy calves by decreasing (−19.4 to −65.6%) the duration and severity of diarrheal episodes [21,24]. On the other hand, the increase observed in the current study for the SI and milk + milk replacer intake could be associated with the presence of *A. paniculata*, *A. indica*, and flavonoids in the evaluated PPA. To support our hypothesis, in the digestive tract of growing lambs, dietary supplementation with a PPA based on *A. paniculata* and *A. indica* decreases the expression of the *Tas2r119* gene [8]. A low expression of the *Tas2r119* gene decreases the production and secretion of anorexigenic molecules, mainly cholecystokinin [29], which could positively impact the SI and milk + milk replacer intake. Furthermore, the flavonoid intake decreases the expression of genes required for the activation of peptide YY receptors in mammalian intestinal tissue [30]. This effect of flavonoids could stimulate the SI and milk + milk replacer intake in dairy calves because peptide YY has anorexigenic effects [17].

On the other hand, the PPA doses (2, 3, and 4 g/d) evaluated in the current study did not affect the FIV between the calves, the FCR, FWH, FHH, or FTG, the number of otitis, pneumonia, and diarrhea cases, or the antibiotic doses applied in the sick calves. The lack of observed changes in the FIV among the calves suggests that the PPA doses tested do not modify welfare in dairy calves [12], while the similarity of the FCR between the treatments indicates that the PPA used does not affect the feed efficiency in dairy calves [8]. On the other hand, the lack of observed changes in the FWH, FHH, and FTG values suggests that the PPA doses tested in the current study do not affect body growth in dairy calves [11]. Furthermore, the results of the present study indicate that the tested treatments cannot be used to decrease the incidence of diseases and antibiotic doses applied in sick dairy calves. Similarly to the current study, recent studies [9,10] have also not detected changes in the FIV between calves or the FCR, FWH, FHH, or FTG in dairy calves supplemented with increasing levels (between 2 and 5 g/d) of different PPAs.

### 4.2. Blood Metabolites

According to Roadknight et al. [31], in dairy calves, the evaluation of hematological parameters is helpful as a diagnostic tool for hematological disorders and some diseases. In the current study, only the blood concentration of segmented neutrophils and plasma protein were modified by PPA supplementation (2 g/d). However, these two and all other hematological parameters evaluated had average values within the reference intervals considered normal in clinically healthy dairy calf populations [31,32]. Therefore, increasing doses (2 to 4 g/d) of the PPA evaluated in the current study could be used as a supplement for dairy calves without affecting their hematological system. Similarly, increasing doses (1, 2, and 3 g/kg DM for 56 days) of the same PPA evaluated in the present study did not affect the hematological parameters in finishing lambs [12]. Likewise, other studies [9,10] showed that supplementation with increasing doses (2 to 5 g/d for 67 days) of other PPAs made mainly with combinations of *A. indica*, *A. paniculata*, and *O. sanctum* also did not affect the average values of hematological parameters in dairy calves.

According to Mohri et al. [33], blood biochemistry parameters are helpful in the diagnosis of renal, hepatic, and nutritional diseases and disorders in dairy calves. In the present study, supplementation with increasing levels (2, 3, and 4 g/d) of PPA decreased the serum glucose concentration. Similarly, recent studies [9,10] also detected lower serum glucose levels in dairy calves supplemented with increasing doses (2, 3, 4, and 5 g/d for 67 days) of some PPAs of a similar composition (based on *A. indica*, *A. paniculata*, and *O. sanctum*) to that evaluated in the current study. According to some authors [9,11], PPAs decrease the serum glucose concentration in dairy calves because they improve the ability to metabolize glucose. On the other hand, the lower serum glucose concentration observed in the present study could be related to hydrolyzable tannins and flavonoids in the tested PPA. This hypothesis is supported by the findings of Brus et al. [34], who observed a lower gene expression of glucose transporters (GLUT2 and GLUT4) in the body tissues of mammals supplemented with hydrolyzable tannins, which led to a lower bioavailability of glucose. Likewise, in young cattle, the intake of flavonoids increases (+35.7 to +78.6%) the serum levels of insulin [35], a hormone that decreases serum glucose levels in dairy calves [36].

In the present study, supplementation with 4 g/d of PPA decreased the serum urea concentration. However, the doses (2, 3, and 4 g/d for 67 days) of PPA evaluated did not affect the serum uric acid and creatinine levels, indicating that the tested PPA does not alter renal function and health in dairy calves [9,10,12]. In the current study, the lack of changes observed in the serum levels of total protein, albumin, globulin, and the albumin/globulin ratio between treatments suggests that the doses of PPA evaluated do not affect protein catabolism in dairy calves [12,21]. On the other hand, the similarity of the serum levels of bilirubin and liver enzymes (alkaline phosphatase, lactate dehydrogenase, and aspartate aminotransferase) observed between the treatments indicates that the evaluated PPA doses do not alter liver function and health in dairy calves [37,38]. Likewise, the lack of changes observed in the serum calcium and phosphorus concentrations suggests that the evaluated PPA does not affect the mineral status in dairy calves [10,31].

## 5. Conclusions

Supplementation with 2 g/d of phytogenic polyherbal additive (ImmuPlus^®^) can be used as a natural alternative to improve the growth rate and stimulate starter and milk + milk replacer intake in dairy calves without adverse effects on health parameters or blood metabolites.

## Figures and Tables

**Table 1:** Growth performance and health parameters of dairy calves supplemented with a polyherbal phytogenic additive ^1^.

	Treatments				SEM	*p*-Value	
Parameter	CON	PPA2	PPA3	PPA4		Linear	Quadratic
Average daily gain (ADG), kg/d	0.672 ^b^	0.783 ^a^	0.750 ^ab^	0.686 ^b^	0.042	0.95	0.02
Final body weight (FBW), kg	86.74 ^b^	93.97 ^a^	91.97 ^ab^	87.62 ^ab^	2.77	0.95	0.02
Milk + replacer intake, liters/d	2.45 ^b^	2.66 ^a^	2.70 ^a^	2.69 ^a^	0.08	0.0001	0.0001
Starter intake (SI), kg/d	0.829 ^b^	0.931 ^a^	0.783 ^b^	0.741 ^b^	0.048	0.16	0.05
Feed intake variation (FIV) among calves, %	107.30	108.50	112.20	115.50	1.13	0.06	0.75
Feed conversion ratio (FCR), kg/kg	2.15	2.03	1.93	2.08	0.02	0.40	0.10
Final wither height (FWH), cm	90.00	91.30	92.00	92.20	3.70	0.07	0.58
Final hip height (FHH), cm	95.40	98.40	97.80	98.60	3.14	0.09	0.51
Final thoracic girth (FTG), cm	101.50	103.50	104.80	101.80	4.44	0.66	0.22
Number diarrheas	0	0	0	0.30	0.15	0.18	0.32
Number pneumonias	3.10	2.00	1.50	1.90	1.04	0.38	0.47
Number otitis	4.20	1.20	2.90	4.90	2.29	0.71	0.28
Antibiotic doses applied in sick calves	5.35	3.38	3.04	5.73	0.43	0.91	0.18

CON: basal diet without polyherbal phytogenic additive (PPA); PPA2: basal diet + 2 g of PPA/kg dry matter (DM); PPA3: basal diet + 3 g of PPA/kg DM; PPA4: basal diet + 4 g of PPA/kg DM. PPA: ^1^ the PPA used was ImmuPlus^®^ (Nuproxa S. de RL. de CV. Querétaro, Mexico), composed of parts of *Whitania somnifera*, *Andrographis paniculata*, *Tinospora cordifolia*, *Ocimum sanctum*, and *Azadirachta indica*. SEM: standard error of the mean. ^a,b^—means within a row with different subscripts differ when *p* ≤ 0.05.

**Table 2:** Hematological profile of dairy calves supplemented with a polyherbal phytogenic additive ^1^.

	Treatments				SEM	*p*-Value	
Parameter	CON	PPA2	PPA3	PPA4		Linear	Quadratic
Hematocrit, %	35.55	36.05	35.35	36.05	0.96	0.85	0.91
Hemoglobin, g/dL	12.06	12.04	11.94	12.25	0.32	0.75	0.61
Red blood cells, 10^12^/L	5.55	5.25	5.37	5.18	0.03	0.22	0.78
Mean corpuscular volume, fL	64.97	68.62	65.93	69.85	0.03	0.19	0.93
Mean corpuscular hemoglobin, pg	22.03	22.93	22.27	23.73	0.03	0.16	0.80
Mean corpuscular hemoglobin concentration, %	33.94	33.39	33.79	33.96	0.21	0.62	0.09
Platelets, 10^9^/L	432.00	464.40	433.30	381.10	35.5	0.25	0.24
Leukocytes, 10^9^/L	10.54	13.00	11.46	14.26	0.08	0.06	0.79
Lymphocytes, %	41.90	39.62	39.80	49.90	3.53	0.10	0.09
Monocytes, %	5.10	3.80	4.30	3.80	0.16	0.25	0.72
Segmented neutrophils, %	46.30 ^b^	53.60 ^a^	48.90 ^ab^	49.60 ^ab^	3.20	0.15	0.03
Band neutrophils, %	3.40	3.00	2.10	3.40	0.17	0.92	0.09
Eosinophils, %	3.00	1.80	4.80	3.20	0.90	0.34	0.85
Basophils, %	0.30	0.10	0.10	0.10	0.11	0.25	0.39
Plasma proteins, g/dL	8.56 ^b^	9.32 ^a^	8.96 ^b^	8.76 ^b^	0.25	0.20	0.04

CON: basal diet without polyherbal phytogenic additive (PPA); PPA2: basal diet + 2 g of PPA/kg dry matter (DM); PPA3: basal diet + 3 g of PPA/kg DM; PPA4: basal diet + 4 g of PPA/kg DM. ^1^ The PPA used was ImmuPlus^®^ (Nuproxa S. de RL. de CV. Querétaro, Mexico), composed of parts of *Whitania somnifera*, *Andrographis paniculata*, *Tinospora cordifolia*, *Ocimum sanctum*, and *Azadirachta indica*. SEM: standard error of the mean. ^a,b^—means within a row with different subscripts differ when *p* ≤ 0.05.

**Table 3:** Biochemistry of blood serum of dairy calves supplemented with a polyherbal phytogenic additive ^1^.

	Treatments				SEM	*p*-Value	
Parameter	CON	PPA2	PPA3	PPA4		Linear	Quadratic
Glucose, mg/dL	71.66 ^a^	64.11 ^b^	56.11 ^c^	53.66 ^d^	1.07	0.01	0.15
β-hydroxybutyrate, mol/L	0.33	0.34	0.40	0.36	0.10	0.40	0.55
Urea, mg/dL	23.44 ^a^	23.22 ^a^	22.55 ^a^	18.33 ^b^	1.62	0.04	0.18
Uric acid, mg/dL	0.46	0.62	0.57	0.54	0.11	0.47	0.09
Creatinin, mg/dL	0.97	0.95	0.86	0.97	0.08	0.91	0.26
Total protein, g/dL	6.97	6.82	6.71	6.75	0.01	0.19	0.44
Albumin, g/dL	4.05	3.82	3.75	3.75	0.15	0.17	0.45
Globulin, g/dL	2.92	3.00	2.95	3.00	0.03	0.57	0.76
Albumin/globulin	1.22	1.27	1.21	1.19	0.06	0.14	0.45
Cholesterol, mg/dL	88.22	84.88	88.88	80.22	3.86	0.25	0.49
Bilirubin, mg/dL	0.35	0.44	0.44	0.44	0.10	0.12	0.33
Aspartate aminotransferase, U/L	23.11	26.66	22.55	22.55	0.10	0.87	0.87
Alkaline phosphatase, U/L	23.33	32.88	31.33	33.22	0.10	0.09	0.19
Lactate dehydrogenase, U/L	79.55	100.44	82.88	77.66	0.07	0.56	0.09
Calcium, mg/dL	9.35	9.43	9.95	9.01	0.34	0.74	0.14
Phosphorus, mg/dL	5.22	4.71	4.71	4.67	0.04	0.11	0.30

CON: basal diet without polyherbal phytogenic additive (PPA); PPA2: basal diet + 2 g of PPA/kg dry matter (DM); PPA3: basal diet + 3 g of PPA/kg DM; PPA4: basal diet + 4 g of PPA/kg DM. ^1^ The PPA used was ImmuPlus^®^ (Nuproxa S. de RL. de CV. Querétaro, Mexico), composed of parts of *Whitania somnifera*, *Andrographis paniculata*, *Tinospora cordifolia*, *Ocimum sanctum*, and *Azadirachta indica*. SEM: standard error of the mean. ^a,b,c,d^—means within a row with different subscripts differ when *p* ≤ 0.05.

## Data Availability

The datasets used and analyzed during the current study are available from the corresponding author on reasonable request. The data are not publicly available due to restrictions on privacy.
